# Moderators and mediators of the relationship between parental depression and children’s emotion dysregulation: a systematic review

**DOI:** 10.3389/fpsyt.2025.1605718

**Published:** 2025-07-08

**Authors:** Ahmad Sabalbal, Samer El Hayek, Evelyne Baroud, Wael Shamseddeen

**Affiliations:** ^1^ Department of Psychiatry, American University of Beirut, Beirut, Lebanon; ^2^ American Center for Psychiatry and Neurology, Dubai, United Arab Emirates

**Keywords:** parental depression, emotion dysregulation, parenting behaviors, parent-child interaction, moderator and mediator

## Abstract

**Background:**

Parental depression is an important risk factor for the development of psychopathology in children/adolescents. Many children who suffer from psychopathology also experience emotion dysregulation, which is characterized by an inability to modulate the intensity and quality of emotions. Emotion dysregulation carries high morbidity and predicts ongoing mood/behavior problems. To develop more effective intervention and prevention programs, it is important to understand the variables that mediate and moderate the relationship between parental depression and children’s emotion dysregulation. This study aimed to systematically explore possible mediators and moderators.

**Methods:**

The PubMed, Scopus, PsycINFO, and Embase databases were systematically searched from day of inception until January 12, 2024. The reference lists of the reviews of interest identified during the screening were included. Two authors screened/collected articles through title and abstract screening, followed by full-text screening. The results were qualitatively synthesized. The inclusion criteria were: *population*, children/adolescents (aged 0–17 years); *exposure*, parental depression; *outcome*, emotion dysregulation; and *study design*, quantitative.

**Results:**

A total of 1,731 studies were identified, of which 556 were potentially eligible. After removing duplicates/retracted articles, 380 records were screened (title/abstract), following which 315 records were excluded. Of the remaining 65 studies, eight met the inclusion criteria after full-text screening. Most of the studies (*n* = 6) included mothers. Biological variables and variables related to the child, to parental depression severity, and to child–parent interactions emerged. The biological variables (the child’s genotype and left parietal alpha asymmetry) highlight a biological vulnerability to dysregulation beyond parent–child effects and environmental factors: left parietal alpha asymmetry was a partial mediator, while genotype was a moderator as children carriers of the S/LG genotypes experienced higher levels of dysregulation as a function of exposure to higher levels of prenatal maternal depression. Depression severity and parent–child dyadic variability were moderators as elevated levels of dysregulation among girls were predicted by greater maternal depression severity and mothers who were more inconsistent in parenting behaviors were more likely to have toddlers with dysregulation, especially if the mothers were depressed. Diet was a mediator, and more severely depressed mothers were more likely to feed their children unhealthy diets, in turn leading to greater dysregulation in later years. Parenting stress mediated the relationship between maternal depression and dysregulation in toddlers.

**Conclusions:**

Children of depressed parents are a vulnerable group and are prone to developing emotion dysregulation. The findings suggest that prevention/intervention programs should target the children of more severely depressed parents and those of parents who engage in more negative interactions with them. Children’s diet and parenting stress are also potential evidence-based, modifiable intervention targets.

**Systematic review registration:**

https://www.crd.york.ac.uk/PROSPERO/, identifier CRD42024502390.

## 1 Introduction

Parental depression is one of the most important risk factors for the development of various forms of psychopathology in children (1–3), and this risk can be transmitted across generations (4–6). The severity and the chronicity of parental depression are strongly associated with more severe internalizing and externalizing problems in children (7, 8). Moreover, parental depression is linked to poorer functioning in children across a range of domains (9).

Emotion regulation is a complex process that involves the ability to identify, evaluate, and manage emotions in a way that is adaptive and socially suitable (10). In children, emotion regulation is influenced by many elements, including their developmental level, neurobiological factors, and social interactions with their parents (11, 12). Emotion dysregulation (ED) is an important transdiagnostic construct characterized by difficulties in regulating the quality and intensity of emotions (13). It is associated with psychopathology, including attention deficit hyperactivity disorder (ADHD), oppositional defiant disorder, personality disorders, self-injurious behaviors, and suicidality (13, 14). ED can affect up to a third of children/adolescents who seek mental health services (15), carries significant morbidity, and increases the risk of developing adult psychiatric disorders (16). Indeed, ED-related behaviors, such as severe tantrums and outbursts, often forecast long-lasting mood/behavior problems and warrant early intervention (12). The relationship between ED and psychopathology is intricate as ED acts both as a mediator and a predictor of outcomes in psychiatric disorders among children/adolescents (13).

While a strong link exists between parental depression and an increased risk of psychiatric disorders in children (2, 3), emerging research is shedding light on the crucial and complex relationship between parental depression and ED in children (17). One recent study found that children of depressed parents exhibit less adaptive emotion regulation strategies compared with children of non-depressed parents (18). While this relationship is likely complex and multifaced, certain parenting behaviors haven been implicated, and parent–child interactions play a significant role in shaping children’s ability to self-regulate (19). Specifically, parental warmth, sensitivity, and proper scaffolding are significantly related to children’s adaptive self-regulation (20, 21). The literature also suggests that depressed parents tend to engage in more negative parenting behaviors (22–24), which may explain how parental depression impacts emotion regulation in children.

Moreover, ED has emerged as an important factor that could contribute to the intergenerational transmission of depression from parents to children, in addition to heritability, intrinsic neurobiology, negative parental behaviors/affect, and child-related stressful events (25). Research indicates that affective and cognitive (emotion regulation) difficulties in children of depressed parents increase their own risk of depression (18), and child dysregulation is a predictor of depression at later developmental time points (26–28).

Given the high-morbidity, transdiagnostic nature of ED and its role in the development of psychopathology, it is important, from an intervention and prevention perspective, to understand what variables mediate and moderate the complex relationship between parental depression and ED in children. This knowledge is, in turn, essential to developing prevention and intervention targets and strategies for children of depressed parents.

Mediators reflect the mechanisms of transmission from parental depression to ED in their children. Identifying these variables helps in determining potential targets of evidence-based interventions, especially early on (29). Moderators define groups of children who are at a higher or a lower risk of experiencing ED due to parental depression (29). Identifying these groups helps in directing interventions to those who need them the most. Mediators therefore highlight pathways that should be targeted by interventions, while moderators help identify who need these interventions.

A recent systematic review and meta-analysis summarized important preventive interventions for children of depressed parents and highlighted the need for more research on preventive intervention. The authors emphasized the importance of understanding which intervention characteristics are the most effective and called for further research into potentially moderating factors (30).

The aims of this study were to systematically explore the possible mediators and moderators of the relationship between parental depression and ED in children. To the best of our knowledge, there are no studies that examined this topic. The results would contribute to the development of effective prevention and intervention strategies.

## 2 Methods

The protocol of this systematic review was registered with the International Prospective Register of Systematic Reviews PROSPERO, registration no. CRD42024502390.

### 2.1 Search strategy

The PubMed, Scopus, PsycINFO, and Embase databases were searched from day of inception until January 12, 2024. No filters were applied, except for the human species filter in PubMed. The search strategy and its relevant MESH terms are outlined in **Supplementary Appendix 1**. A medical librarian experienced in systematic review searches was consulted for the design of the search strategy. The search included the reference lists of reviews of interest identified during the preliminary screening process. The authors also hand-searched the reference lists of the final included studies and used Google Scholar to find the relevant articles that cited these studies.

### 2.2 Eligibility criteria

The inclusion criteria were as follows:

Population: Children and adolescents (0–17 years)
Exposure: Parental depression (maternal/paternal)
Outcomes: ED in children/adolescents
Study design: Quantitative studies including cross-sectional, case–control, cohort, and randomized controlled trials

### 2.3 Exclusion criteria

Studies on the following were excluded:

Population: Youth older than 18 years
Exposure: Other psychiatric disorders in parents (e.g., psychotic disorders, substance use disorders, trauma-related disorders, personality disorders, and others)
Outcomes: Outcomes other than ED in children/adolescents
Design: Literature reviews, systematic studies, and epidemiological and qualitative manuscripts

### 2.4 Assessment of study quality

To evaluate the methodological quality and risk of bias, the National Institutes of Health (NIH) Quality Assessment Tool for Observational Cohort and Cross-Sectional Studies was used (31).

### 2.5 Study selection process

EndNote software version 21 was used to import all studies obtained from the literature search. After removing duplicate references, the articles were screened by two different investigators who conducted an independent title and abstract screening using a standardized screening guide. Disagreements were resolved by including the article in the full-text screening stage. The title and abstract screening steps were followed by an independent full-text screening performed by the same investigators using a standardized screening guide. Disagreements were resolved through discussion.

### 2.6 Data abstraction

The included studies were screened by two investigators who independently extracted and compared the data of interest from the full texts. For each study, the investigators obtained the following information: the year of publication; characteristics of the children/adolescents [e.g., sample size, age, length of follow-up, measure of ED (i.e., scales used to assess the outcome)]; characteristics of the parents (e.g., sample size, age, and characteristics of depression including the method of assessment, duration, and severity, when applicable); and the moderators and mediators. The authors also collected information about funding and conflict of interest.

### 2.7 Definition of moderators and mediators

Moderators were defined as baseline variables that had a significant statistical interaction with parental depression in predicting ED in children in cohort or longitudinal studies. Mediator variables were required to occur chronologically between the parental depression (the exposure) and the ED (the outcome). Studies investigating mediators were required to include at least three time points. In addition, a variable was considered a mediator if it met the following criteria: 1) it was significantly associated with parental depression; 2) it was significantly associated with the outcome; and 3) upon controlling for the mediator, the effect size of the association between parental depression and the outcome decreased (partial mediators) or lost significance (full mediator). Given the variability in the control variables across the studies, it was decided that separating between partial and full mediators might not be very meaningful.

### 2.8 Data synthesis

Given the heterogeneous nature of the included studies, a qualitative synthesis of the results was conducted.

## 3 Results

### 3.1 PRISMA flow results

**Figure 1:**
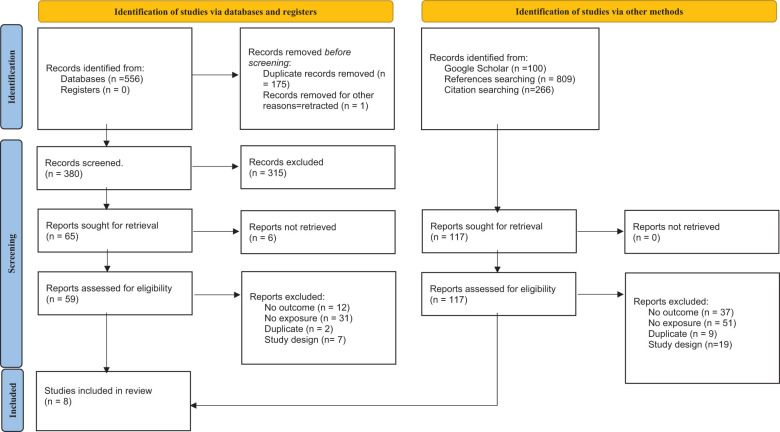
PRISMA flow diagram.

A total of 556 potentially eligible studies were identified following an electronic search of the four databases. After removing duplicates and retracted articles, 380 records were screened by title and abstract. A total of 315 records were excluded. Of the remaining 65 articles, 58 were further excluded after full-text screening, as outlined in **Figure 1** as per the guidelines (32). After checking the references and citations of the included studies, one additional relevant study was retrieved and included. The final list of articles comprised eight studies that address the three above-mentioned themes.

### 3.2 Characteristics of the included studies

The main findings of the included studies, including the study characteristics, sample sizes, follow-up duration, and the measures used, are summarized in **Table 1**. **Supplementary Appendix 2** provides detailed information about each study and the extracted data. Most of the studies (*n* = 6) assessed the effect of maternal depression on children’s/adolescents’ ED. Two studies examined the effect of paternal depression (33, 34).

**Table 1:** Summary of the characteristics of the included studies.

Study (author, year)	Parent characteristics			Children characteristics		Study type and follow-up duration (years)	Measurement tools for children’s emotion dysregulation	Risk of bias assessment
	Initial no. of participants and parent gender	Timing of depression assessment	Parent depression tool	No. of participants at the end of the study	Child’s age at outcome (ED) assessment			
Babineau et al., 2015 (35)	213 dyads Mothers	Twice prenatally (gestation age, 24–36 weeks) Four times postnatally (6, 12, 24, and 36 months)	Center for Epidemiological Studies—Depression (CES-D)	213	3, 6, 18, and 36 months	Longitudinal (3)	The Infant Behavior Questionnaire—Revised (IBQ-R) Early Childhood Behavioral Questionnaire (ECBQ)	Good
Felton et al., 2021 (36)	206 dyads Mothers	Every time point T15 through T18 At T15, the mother’s average age was 46 years (range, 30–59 years)	Center for Epidemiological Studies—Depression (CES-D)	206 at T15 177 at T16 153 at T17 151 at T18	9–13 years at initial assessment Range of 13–17 years (average, 15) at baseline Outcome assessed at ages 13–17 years	Longitudinal (4)	The Behavioral Indicator of Resiliency to Distress (BIRD)	Good
Fredriksen et al., 2019 (33)	1,036 Mothers and fathers	Prenatal data collection waves (T1–T3): average gestational week 21 (T1), week 28 (T2), week 32 (T3), and week 36 (T4) Postnatal data collection waves (T5–T7): child age 6 weeks postpartum (T5), 6 months postpartum (T6), 12 months postpartum (T7), and 18 months postpartum (T8)	Edinburgh Postnatal Depression Scale (EPDS)	387 mothers 32 fathers 162 father–mother	18 months	Longitudinal (2)	The Infant–Toddler Social and Emotional Assessment (ITSEA)	Good
Gonzalez, 2009 (40)	132 dyads Mothers	At infant’s age 20 months	Diagnostic Interview Schedule	132	48 months	Longitudinal (1.33)	Tool use and Problem-Solving Task	Poor
Hoffman, 2006 (37)	208 Mothers and fathers	Children aged between 30 and 36 months	Center for Epidemiological Studies—Depression (CES-D)	208	47–49 months	Longitudinal (1)	Dysregulation Coding System	Fair
Lunkenheimer et al., 2021 (34)	100 Mothers and fathers	At child’s age 3.5 years	Center for Epidemiological Studies—Depression (CES-D)	100	45 months	Longitudinal (0.33)	Emotion Regulation Checklist	Fair
Marino et al., 2019 (39)	104 dyads Mothers	Postnatally, 2 weeks prior to child’s age of 6 months	Achenbach System of Empirically Based Assessment	104	24 months	Longitudinal (2)	Child Behavior Checklist—Dysregulation Profile	Good
Pina-Camacho et al., 2015 (38)	7,814 mother–child pairs	Twice prenatally (18 and 32 weeks of gestation), and four times postnatally (8 weeks, 8 months, 2 years, and 3 years)	Edinburg Postnatal Depression Scale (EPDS)	7,814	7 years old	Longitudinal (7)	Strengths and Difficulties Questionnaire (SDQ-DP)	Good

*ED*, emotion dysregulation.

The included studies were mostly longitudinal, with sample sizes ranging from 66 to 7,814 parent–child pairs and follow-up periods reaching up to 4 years. The age range of children varied between 6 months and 7 years. A variety of tools and instruments were used to measure the constructs of interest. Parental depression was assessed using four different scales/measures. Four studies used the Center for Epidemiological Studies—Depression (34–37), while two used the Edinburgh Postnatal Depression Scale, specifically evaluating maternal postnatal depression (33, 38). The Achenbach system of empirically based assessment was used in one study (39), and one study included mothers with a history of depression, without specifying a screening tool (40).

ED was assessed using a range of instruments, including the Strengths and Difficulties Questionnaire, Dysregulation Profile (SDQ-DP); the Child Behavior Checklist, Dysregulation Profile (CBCL-DP); and other experimental methods to assess dysregulation in younger populations (e.g., Behavioral Indicator of Resiliency to Distress Tool Use and Problem-Solving Task and the Infant–Toddler Social and Emotional Assessment). The timing of parental depression and the age of the children at outcome evaluation varied across the studies.

There were five included studies that were rated good quality, two were assessed as fair, and one was determined to be of poor quality.

### 3.3 Moderators

Moderators were categorized into three meaningful categories: 1) biological variables; 2) variables related to parental depression severity; and 3) variables related to child–parent interactions.

1. Biological variables

One study investigated the effect of child genotype as a moderator of the relationship between prenatal maternal depression and child dysregulation profile at the ages of 3, 6, 18, and 36 months (35). There was a significant effect of the child serotonin transporter gene (5-HTT) and a polymorphic serotonin transporter-linked polymorphic region (5-HTTLPR). While carriers of the L_A_L_A_ genotype were insensitive to prenatal depression exposure (i.e., had stable scores of dysregulation across follow-ups), children who were carriers of the S/L_G_ genotype experienced higher levels of dysregulation as a function of exposure to higher levels of prenatal depression. When exposed to lower levels of maternal prenatal depression, S/L_G_ carriers experienced lower levels of dysregulation than did L_A_ carriers (35).

2. Severity of parental depression

Two studies examined parental depression severity. One analyzed 206 mother–children dyads at five time points (36). At the starting point (wave 4), the average age of adolescents was 15 years (range, 13–17 years). On yearly follow-up over 4 years, the baseline severity of maternal depressive symptoms moderated the impact of depression on ED, whereby higher levels of ED among girls were predicted by higher baseline levels of maternal depression. In this study, distress tolerance was used as a proxy for ED, and the results pointed toward maternal depressive symptoms as shaping girls’ ability to tolerate distress (36).

The other study included 1,036 families, followed up at seven time intervals (prenatally and postnatally). Parental depression severity did not emerge as a moderator (33).

3. Parent–child interaction variables

One study investigated parent–child dyadic variability (dyadic behavioral variability, DBV) as a potential moderating variable (34). DBV refers to the variability of dyadic interaction patterns, specifically the range of emotional states of the dyads observed during a parent–child interaction (41). A sample of 100 parents and 3.5-year-old children participated and were assessed at baseline (T1), then 4 months later (T2). ED was assessed through maternal report of the child’s emotional negativity/lability on the Emotion Regulation Checklist. Mothers who were more inconsistent in their behaviors (shifting between different parenting styles) were more likely to have toddlers with difficulty controlling their emotions, especially if the mothers were experiencing depression (34).

### 3.4 Mediators

Mediators were classified into three categories: 1) biological variables; 2) child-related variables; and 3) variables related to child–parent interactions and parenting.

1. Biological factors

One study examined the electroencephalogram (EEG) alpha asymmetry patterns of infants at the age of 6 months as a potential mediator of dysregulation when children were 24 months old. Maternal depression (measured 2 weeks before the infant’s 6-month birthday) was the exposure (39). Left parietal alpha asymmetry (PAA) partially mediated the effect of maternal depression on ED in children, while frontal alpha asymmetry (FAA) did not show a significant mediating role (39).

2. Child-related variables

In a longitudinal study of 7,814 mother–offspring pairs, maternal depression was assessed at two time points prenatally (18 and 32 weeks of gestation) and four times postnatally (8 weeks, 8 months, 2 years, and 3 years) (38). ED was evaluated at children’s ages of 2, 4, and 7 years using the SDQ-DP, with the main outcome being ED at 7 years. The results showed that children who ate more processed foods and sugary snacks (such as chips, cookies, and candy) were more likely to have trouble controlling their emotions later in life, especially if their mothers had experienced depression during pregnancy (38). Mothers with higher levels of depression during pregnancy were more likely to feed their children unhealthy diets at the age of 3 years. This, in turn, led to greater levels of dysregulation when the child was 7 years old (38).

3. Variables related to child–parent interactions and parenting

Gonzalez evaluated children of mothers with a history of depression at the age of 20 months (time 1), 36 months (time 2), and 48 months (time 3). Children were subjected to the “strange situation” at times 1 and 2. There were two mediators of interest: maternal affective discourse and attachment insecurity. Although these variables were found to be predictors of dysregulation, there were no significant direct effects of maternal depression on the ED of toddlers (as measured by Emotional Lability/Being Disengaged at time 3), thereby ruling them out as potential mediators (40).

One study evaluated parents for depression at several time points both prenatally and postnatally and used the Parent Stress Index to measure parenting stress when the children were 12 months old (33). Parenting stress was a broad mediator between parents’ postnatal depressive symptoms and various child outcomes. Specifically, parenting stress mediated the relationship between maternal dysphoria and both externalizing and dysregulation problems in the children, and it mediated the relationship between paternal postnatal depressive symptoms and receptive communication when the children were 18 months old (33).

Lastly, one study (37) examined maternal scaffolding as a variable using a sample of approximately 200 children and their parents, followed up for 1 year (at the start of the study, the children were 3 years old). Children between 35 and 39 months old underwent a laboratory task that would allow evaluating maternal scaffolding. Effective scaffolding happened when the mother provided a level of support that allowed her child to succeed at the task at hand, beyond what the child would have been able to accomplish alone. ED was later measured when the children were between the ages of 47 and 49 months, again in the laboratory using three problem-solving tasks (problem-solving tasks, clean-up task, and wait task). There was no significant mediating role for ineffective maternal scaffolding.

## 4 Discussion

This study systematically reviewed moderators and mediators of the relationship between parental depression and ED in children. The child’s 5-HTTLPR genotype, the severity of parental depression, and the parent–child DBV were significant moderators. The child’s unhealthy diet at age 3 years, parenting stress, and infant EEG alpha asymmetry were significant mediators.

The genetic correlates of ED in the pediatric population remain relatively understudied. However, the finding of the child’s 5-HTTLPR polymorphism as a moderator is interesting and aligns with the existing literature on the genetic underpinnings of ED (42). The serotonin transporter gene (5-HTT) and a polymorphic serotonin transporter-linked polymorphic region modulate serotonin synaptic signaling during neurotransmission (43). There are three variants of 5-HTTLPR alleles: the short (S) allele, the long-rs25531(G) (La) allele, and the long-rs25531(A) (La) allele. Compared with the (La) allele variant carriers, the short (S) allele and the long (Lg) allele express lower mRNA transcriptions of the serotonin transporter (44). This polymorphism appears to be correlated with the severity of mood dysregulation and affective reactivity (45). A meta-analysis has indeed shown that 5-HTTLPR polymorphism is responsible for 10% of the variance in amygdala activation (46), a key brain region involved in the processing of emotions (47). Children who are carriers of S alleles exhibit increased amygdala during processing of emotionally negative stimuli compared with individuals with homozygous L alleles (48). It is worth noting that this genetic vulnerability to ED appears to be susceptible to the moderating and mediating influences of the environment (49).

Other genetic factors have been identified in relation to emerging emotion regulation problems in youth. For instance, one study showed that carriers of the Val158Met polymorphism of the catechol-*O*-methyltransferase (*COMT*) gene, which regulates frontal dopaminergic activity, tend to be more impulsive in decision-making than non-carriers (50). Another study found that the DRD2 Taq A1 allele of the dopamine receptor gene is associated with increased sensitivity and emotional responsiveness to negative feedback while simultaneously reducing sensitivity to positive feedback (51). To our knowledge, these studies did not incorporate the impact of parental mental health in their work. Depression characteristics such as severity, duration, and timing may play a role in the context of these genetic factors. Therefore, a better understanding of the interplay between parental depression and genetic vulnerability to dysregulation in children is a critical area for future research. The study identified in this review emphasizes the early impact of maternal depression on children who carry the S/LG genotypes. These children exhibited higher levels of dysregulation as a function of exposure to greater levels of prenatal depression. Consequently, interventions tailored to this population at a young age may be a promising area for future investigation.

Another biological variable identified was the infant’s PAA on EEG, which highlights a biological vulnerability to dysregulation beyond mother–child effects and environmental factors (39). Interestingly, PAA is also found among children with ADHD who experience ED (52). Further exploration of the genetic and biological background of ED is an important area of research in order to identify biomarkers and to develop tailored and personalized treatments for children and adolescents with mood disorders (42).

Two studies examined the potential moderating role of parental depression, but only one study found a statistically significant moderating effect. Although both studies assessed participants at several time points, the follow-up durations differed (4 *vs*. 2 years.), which may have accounted for the differences in the findings. Another potential explanation is that the youth were of different ages when the outcome was measured (teenagers compared to toddlers). It is well established that the severity and the chronicity of parental depression are correlated with more severe internalizing and externalizing problems in children (8), and given the association between ED and psychopathology (13), it is not surprising that maternal depression severity emerged as a moderator of dysregulation during adolescence. Adolescence is indeed a critical period for social and emotional development, and the parent–adolescent relationship specifically shapes adolescents’ social and emotional wellbeing (53). For example, adolescents who struggle to communicate with their parents about their difficulties are more likely to experience emotional/behavioral difficulties (54), and elevated levels of parent–teen conflict, including hostility, are associated with externalizing and internalizing disorders (55). Mothers who experience more severe depression may therefore engage in more negative interactions with their teenagers, potentially contributing to increased ED. Consistent with these findings, one of the included studies found that mothers’ parenting stress mediated the relationship between postnatal depression and child dysregulation. This finding is in line with the existing literature, which links parenting stress to child behavior problems (56). Notably, this study assessed parents when their children were very young (12 months old). This highlights the importance of evaluating parenting stress as part of the routine care of young children and during the evaluation and treatment of depressed parents of young children.

There is strong evidence in the literature suggesting that parenting factors, such as parental rejection and control, contribute to the relationship between parental depression and subsequent youth psychopathology (23, 24). This review found that DBV was associated with greater dysregulation among toddlers when their parents exhibited high levels of depression, emphasizing the significance of parent–child interactions across developmental years. The early years lay the stage for later emotion regulation, and the caregiver–child interaction is an important starting point as it influences children’s mental health. Positive caregiver–child relationships improve the emotional security and self-esteem of the child and promote the development of social skills and adaptive emotion regulation strategies (57). It is therefore important to consider parental depression whenever a child is experiencing deficits in emotion regulation strategies, as the root of their difficulties may be related to the parent rather than the child. It is equally important to be mindful of depressive symptoms in these children as negative interactions with parents appear to be more common among depressed preschoolers compared with healthy ones (58). While the study by Gonzalez did not find maternal affective discourse to act as a mediator, it did uncover an association between maternal depression and maternal availability (40), whereby mothers experiencing depression were less likely to be emotionally available or to promote positive affect toward their child. Research has shown that maternal emotional unavailability is associated with poor emotion regulation in children (59). One potential explanation for the results in Gonzalez may be the relatively short follow-up time and the impact of maternal affective discourse may not have been apparent on such a young population.

One of the included studies did not find a mediating effect for toddlers’ attachment insecurity. However, there was an indirect effect, whereby higher attachment insecurity in toddlers at 36 months predicted dysregulation at the age of 48 months. While this finding does not qualify as mediation, it highlights the role of a child’s attachment in shaping their regulation strategies. Research suggests that disorganized/controlling attachment, rather than insecure attachment, is correlated with maternal depression (60).

The finding that an unhealthy diet mediated the relationship between maternal depression and the subsequent ED is particularly noteworthy as it highlights the importance of considering parental care beyond parenting behaviors and extends to nutrition/diet. Studies examining the physical health of the children of depressed mothers suggest that maternal depression, specifically during the postpartum period, appears to be associated with more frequent acute child healthcare (emergency visits) than regular follow-up clinic visits (61). Furthermore, compared with infants of mothers who are not depressed, those of depressed mothers are more likely to experience illness, pain, and higher morbidity and mortality (62). Poor dietary habits can therefore be viewed as a component of maternal care that influences the health of a child. Incorporating education about the importance of diet during prenatal or postnatal care and screening for dietary habits in the children of depressed parents may be important for the prevention of ED. Indeed, a recent systematic review has highlighted a consistent cross-sectional association between unhealthy dietary patterns and worse mental health in childhood and adolescence (63), and one interesting pilot study on micronutrient treatment for children with emotional and behavioral dysregulation showed behavioral improvement through better communication with parents and less impulsivity (64).

This systematic review has a number of limitations. It was not possible to conduct a meta-analysis due to the heterogeneity of the included studies. Despite a comprehensive search strategy, only eight studies met the inclusion criteria after full screening. Because of the small number of studies, the obtained results cannot be generalized to various settings or across different cultural and socioeconomic backgrounds and may limit the strength of our conclusions. Due to the absence of gender-based breakdown of the results, we are unable to elaborate on a potential influence of children’s biological sex on the relationship between parental depression and children’s dysregulation. One of the included studies (36) incorporated an analysis of gender, with the results showing a differential impact of parental depression in girls (36). In general, women are more likely to experience internalizing problems such as anxiety and depression, while men tend to present with externalizing problems such as aggression and impulsivity (65). This distinct susceptibility to types of emotional and behavioral problems in women and men are potentially linked to the different forms of ED and may also have a differential impact on parental perceived self-efficacy, a key predictor of positive and successful parenting (66). There is therefore a need for more focused studies on the role of children’s gender in this relationship. Lastly, six out of the eight included studies focused exclusively on mothers, further limiting the generalizability of the findings to fathers. Paternal depression may entail distinct outcomes for children or involve different mechanisms in influencing children’s emotion regulation, and there is a need for more research on fathers.

In conclusion, this systematic review is, to our knowledge, the first to examine mediators and moderators of the relationship between parental depression and ED in children. The findings highlight the importance of focusing on the children of depressed parents as a vulnerable group who are at increased risk of developing ED. Parental depression severity, diet, and parenting stress emerged as important variables. Further research is needed to evaluate specific parenting factors as potential mediators. Future research should also explore how cultural and societal values and characteristics influence youth emotion regulation, which is important for the development of adequately tailored interventions.

## Data Availability

The original contributions presented in the study are included in the article/**Supplementary Material**. Further inquiries can be directed to the corresponding author.
