# Supplementary material for: Moderators and mediators of the relationship between parental depression and children’s emotion dysregulation: a systematic review
Source: Front Psychiatry. 2025 Jul 8;16:1605718. doi: 10.3389/fpsyt.2025.1605718 (PMC12279834; doi:10.3389/fpsyt.2025.1605718)
Supplement: Supplementary file 1 [file Supplementaryfile1.docx]

| Mesh | Synonyms |
| --- | --- |
| “Parents” [**MeSH Terms]** | Parent [Title/Abstract] OR  Parenthood Status [Title/Abstract] OR  Status, Parenthood [Title/Abstract] OR  Parental [Title/Abstract] OR  Parents [Title/Abstract] OR |
| “Mothers” [**MeSH Terms]** | Mother [Title/Abstract] OR  Mothers [Title/Abstract] OR |
| “Fathers” [**MeSH Terms]** | Father [Title/Abstract] OR  Fathers [Title/Abstract] OR |
|  | Paternal [Title/Abstract] OR |
|  | Maternal [Title/Abstract] OR |

**Appendix 1**. Search Strategy & mesh terms

1.PUBMED

Parents

Depression

| Mesh terms | Synonyms |
| --- | --- |
| “Depression” [**MeSH Terms]** | “Depression” [Title/Abstract] OR |
| “Depression, Postpartum” [**MeSH Terms]** | “Depression, Postpartum” [Title/Abstract] OR  “Postnatal Depression” [Title/Abstract] OR  “Depression, Postnatal” [Title/Abstract] OR  “Post-Partum Depression” [Title/Abstract] OR  “Depression, Post-Partum” [Title/Abstract] OR  “Post Partum Depression” [Title/Abstract] OR  “Postpartum Depression” [Title/Abstract] OR  “Post-Natal Depression” [Title/Abstract] OR  “Post Natal Depression” [Title/Abstract] OR  “Postnatal Dysphoria” [Title/Abstract] OR  “Post-Partum Dysphoria” [Title/Abstract] OR  “Post Partum Dysphoria” [Title/Abstract] OR  “Postpartum Dysphoria” [Title/Abstract] OR  “Dysphoria, Postpartum” [Title/Abstract] OR  “Post-Natal Dysphoria” [Title/Abstract] OR  “Post Natal Dysphoria” [Title/Abstract] OR |
| “Depressive Disorder” [**MeSH Terms]** | “Depressive Disorder” [Title/Abstract] OR  “Depressive Disorders” [Title/Abstract] OR  “Disorder, Depressive” [Title/Abstract] OR  “Disorders, Depressive” [Title/Abstract] OR  “Neurosis, Depressive” [Title/Abstract] OR  “Depressive Neuroses” [Title/Abstract] OR  “Depressive Neurosis” [Title/Abstract] OR  “Neuroses, Depressive” [Title/Abstract] OR  “Depression, Endogenous” [Title/Abstract] OR  “Depressions, Endogenous” [Title/Abstract] OR  “Endogenous Depression” [Title/Abstract] OR  “Endogenous Depressions” [Title/Abstract] OR  “Depressive Syndrome” [Title/Abstract] OR  “Depressive Syndromes” [Title/Abstract] OR  “Syndrome, Depressive” [Title/Abstract] OR  “Syndromes, Depressive” [Title/Abstract] OR  “Depression, Neurotic” [Title/Abstract] OR  “Neurotic Depression” [Title/Abstract] OR  “Neurotic Depressions” [Title/Abstract] OR  “Melancholia” [Title/Abstract] OR  “Melancholias” [Title/Abstract] OR  “Unipolar Depression” [Title/Abstract] OR  “Depression, Unipolar” [Title/Abstract] OR  “Depressions, Unipolar” [Title/Abstract] OR  “Unipolar Depressions” [Title/Abstract] OR |
| “Depressive Disorder, Major” [**MeSH Terms]** | “Depressive Disorder, Major” [Title/Abstract] OR  “Depressive Disorders, Major” [Title/Abstract] OR  “Major Depressive Disorders” [Title/Abstract] OR  “Major Depressive Disorder” [Title/Abstract]OR  “Depression, Involutional” [Title/Abstract] OR  “Involutional Depression” [Title/Abstract] OR  “Involutional Melancholia” [Title/Abstract] OR |

Child

| Mesh terms | Synonyms |
| --- | --- |
| “Adolescent” [**MeSH Terms]** | “Adolescent” [Title/Abstract] OR  “Adolescents” [Title/Abstract] OR  “Adolescence” [Title/Abstract] OR  “Teens” [Title/Abstract] OR  “Teen” [Title/Abstract] OR  “Teenagers” [Title/Abstract] OR  “Teenager” [Title/Abstract] OR  “Youth” [Title/Abstract] OR  “Youths” [Title/Abstract] OR  “Adolescents, Female” [Title/Abstract] OR  “Adolescent, Female” [Title/Abstract] OR  “Female Adolescent” [Title/Abstract] OR  “Female Adolescents” [Title/Abstract] OR  “Adolescents, Male” [Title/Abstract] OR  “Adolescent, Male” [Title/Abstract] OR  “Male Adolescent” [Title/Abstract] OR  “Male Adolescents” [Title/Abstract] OR |
| “Psychology, Adolescent” [**MeSH Terms]** | “Psychology, Adolescent” [Title/Abstract] OR  “Adolescent Psychology” [Title/Abstract] OR |
| “Adolescent Psychiatry” [**MeSH Terms]** | “Adolescent Psychiatry” [Title/Abstract] OR  “Psychiatry, Adolescent” [Title/Abstract] OR |
| “Child Psychiatry” [MeSH Terms] | “Child Psychiatry” [Title/Abstract] OR  “Psychiatry, Child” [Title/Abstract] OR |
| “Adolescent Behavior” [MeSH Terms] | “Adolescent Behavior” [Title/Abstract] OR  “Behavior, Adolescent” [Title/Abstract] OR  “Adolescent Behaviors” [Title/Abstract] OR  “Behaviors, Adolescent” [Title/Abstract] OR |
| “Child” [**MeSH Terms]** | “Child” [Title/Abstract] OR  “Children” [Title/Abstract] OR |
| “Minors” [**MeSH Terms**] | “Minor” [Title/Abstract] OR  “Minors” [Title/Abstract] OR |
|  |  |

Emotional Dysregulation

| No mesh terms | “Emotional dysregulation” [Title/Abstract] OR |
| --- | --- |
|  | “Emotion dysregulation” [Title/Abstract] OR |

1. EMBASE

(((‘parents’ OR ‘parent’ OR ‘parenthood status’ OR ‘status parenthood’ OR ‘parental’ OR ‘mothers’ OR ‘mother’ OR ‘fathers’ OR ‘father’ OR ‘paternal’ OR ‘maternal’) AND (‘depression’ OR ‘depression postpartum’ OR ‘Postnatal Depression’ OR ‘depression postnatal’ OR ‘post partum depression’ OR ‘depression post partum’ OR ‘post partum depression’ OR ‘Postpartum Depression’ OR ‘post natal depression’ OR ‘post natal depression’ OR ‘Postnatal Dysphoria’ OR ‘post partum dysphoria’ OR ‘post partum dysphoria’ OR ‘Postpartum Dysphoria’ OR ‘dysphoria postpartum’ OR ‘post natal dysphoria’ OR ‘post natal dysphoria’ OR ‘Depressive Disorder’ OR ‘Depressive Disorders’ OR ‘disorder depressive’ OR ‘disorders depressive’ OR ‘neurosis depressive’ OR ‘Depressive Neuroses’ OR ‘Depressive Neurosis’ OR ‘neuroses depressive’ OR ‘depression endogenous’ OR ‘depressions endogenous’ OR ‘Endogenous Depression’ OR ‘Endogenous Depressions’ OR ‘Depressive Syndrome’ OR ‘Depressive Syndromes’ OR ‘syndrome depressive’ OR ‘syndromes depressive’ OR ‘depression neurotic’ OR ‘Neurotic Depression’ OR ‘Neurotic Depressions’ OR ‘Melancholia’ OR ‘Melancholias’ OR ‘Unipolar Depression’ OR ‘depression unipolar’ OR ‘depressions unipolar’ OR ‘Unipolar Depressions’ OR ‘depressive disorder major’ OR ‘depressive disorders major’ OR ‘Major Depressive Disorders’ OR ‘Major Depressive Disorder’ OR ‘depression involutional’ OR ‘Involutional Depression’ OR ‘Involutional Melancholia’)) AND ((‘Adolescent’ OR ‘Adolescents’ OR ‘Adolescence’ OR ‘Teens’ OR ‘Teen’ OR ‘Teenagers’ OR ‘Teenager’ OR ‘Youth’ OR ‘Youths’ OR ‘adolescents female’ OR ‘adolescent female’ OR ‘Female Adolescent’ OR ‘Female Adolescents’ OR ‘adolescents male’ OR ‘adolescent male’ OR ‘Male Adolescent’ OR ‘Male Adolescents’ OR ‘psychology adolescent’ OR ‘Adolescent Psychology’ OR ‘Adolescent Psychiatry’ OR ‘psychiatry adolescent’ OR ‘Child Psychiatry’ OR ‘psychiatry child’ OR ‘Adolescent Behavior’ OR ‘behavior adolescent’ OR ‘Adolescent Behaviors’ OR ‘behaviors adolescent’ OR ‘Child’ OR ‘Children’ OR ‘Minor’ OR ‘Minors’) AND (‘Emotional dysregulation’ OR ‘Emotion dysregulation’))) AND ‘human’/de

3.PSYCINFO

| **S3** | #S1 AND #S2 |
| --- | --- |
| **S2** | Any field:  (Adolescent OR Adolescents OR Adolescence OR Teens OR Teen OR Teenagers OR Teenager OR Youth OR Youths OR adolescents female OR adolescent female OR Female Adolescent OR Female Adolescents OR adolescents male OR adolescent male OR Male Adolescent OR Male Adolescents OR psychology adolescent OR Adolescent Psychology OR Adolescent Psychiatry OR psychiatry adolescent OR Child Psychiatry OR psychiatry child OR Adolescent Behavior OR behavior adolescent OR Adolescent Behaviors OR behaviors adolescent OR Child OR Children OR Minor OR Minors) AND (Emotional dysregulation OR Emotion dysregulation) |
| **S1** | Any field:  (parents OR parent OR parenthood status OR status parenthood OR parental OR mothers OR mother OR fathers OR father OR paternal OR maternal) AND (depression OR depression postpartum OR Postnatal Depression OR depression postnatal OR post partum depression OR depression post partum OR post partum depression OR Postpartum Depression OR post natal depression OR post natal depression OR Postnatal Dysphoria OR post partum dysphoria OR post partum dysphoria OR Postpartum Dysphoria OR dysphoria postpartum OR post natal dysphoria OR post natal dysphoria OR Depressive Disorder OR Depressive Disorders OR disorder depressive OR disorders depressive OR neurosis depressive OR Depressive Neuroses OR Depressive Neurosis OR neuroses depressive OR depression endogenous OR depressions endogenous OR Endogenous Depression OR Endogenous Depressions OR Depressive Syndrome OR Depressive Syndromes OR syndrome depressive OR syndromes depressive OR depression neurotic OR Neurotic Depression OR Neurotic Depressions OR Melancholia OR Melancholias OR Unipolar Depression OR depression unipolar OR depressions unipolar OR Unipolar Depressions OR depressive disorder major OR depressive disorders major OR Major Depressive Disorders OR Major Depressive Disorder OR depression involutional OR Involutional Depression OR Involutional Melancholia) |

4.SCOPUS

((("parents"[ALL] OR "parent"[ALL] OR "parenthood status"[ALL] OR "status parenthood"[ALL] OR "parental"[ALL] OR "mothers"[ALL] OR "mother"[ALL] OR "fathers"[ALL] OR "father"[ALL] OR "paternal"[ALL] OR "maternal"[ALL]) AND ("depression"[ALL] OR "depression postpartum"[ALL] OR "Postnatal Depression"[ALL] OR "depression postnatal"[ALL] OR "post partum depression"[ALL] OR "depression post partum"[ALL] OR "post partum depression"[ALL] OR "Postpartum Depression"[ALL] OR "post natal depression"[ALL] OR "post natal depression"[ALL] OR "Postnatal Dysphoria"[ALL] OR "post partum dysphoria"[ALL] OR "post partum dysphoria"[ALL] OR "Postpartum Dysphoria"[ALL] OR "dysphoria postpartum"[ALL] OR "post natal dysphoria"[ALL] OR "post natal dysphoria"[ALL] OR "Depressive Disorder"[ALL] OR "Depressive Disorders"[ALL] OR "disorder depressive"[ALL] OR "disorders depressive"[ALL] OR "neurosis depressive"[ALL] OR "Depressive Neuroses"[ALL] OR "Depressive Neurosis"[ALL] OR "neuroses depressive"[ALL] OR "depression endogenous"[ALL] OR "depressions endogenous"[ALL] OR "Endogenous Depression"[ALL] OR "Endogenous Depressions"[ALL] OR "Depressive Syndrome"[ALL] OR "Depressive Syndromes"[ALL] OR "syndrome depressive"[ALL] OR "syndromes depressive"[ALL] OR "depression neurotic"[ALL] OR "Neurotic Depression"[ALL] OR "Neurotic Depressions"[ALL] OR "Melancholia"[ALL] OR "Melancholias"[ALL] OR "Unipolar Depression"[ALL] OR "depression unipolar"[ALL] OR "depressions unipolar"[ALL] OR "Unipolar Depressions"[ALL] OR "depressive disorder major"[ALL] OR "depressive disorders major"[ALL] OR "Major Depressive Disorders"[ALL] OR "Major Depressive Disorder"[ALL] OR "depression involutional"[ALL] OR "Involutional Depression"[ALL] OR "Involutional Melancholia"[ALL])) AND (("Adolescent"[ALL] OR "Adolescents"[ALL] OR "Adolescence"[ALL] OR "Teens"[ALL] OR "Teen"[ALL] OR "Teenagers"[ALL] OR "Teenager"[ALL] OR "Youth"[ALL] OR "Youths"[ALL] OR "adolescents female"[ALL] OR "adolescent female"[ALL] OR "Female Adolescent"[ALL] OR "Female Adolescents"[ALL] OR "adolescents male"[ALL] OR "adolescent male"[ALL] OR "Male Adolescent"[ALL] OR "Male Adolescents"[ALL] OR "psychology adolescent"[ALL] OR "Adolescent Psychology"[ALL] OR "Adolescent Psychiatry"[ALL] OR "psychiatry adolescent"[ALL] OR "Child Psychiatry"[ALL] OR "psychiatry child"[ALL] OR "Adolescent Behavior"[ALL] OR "behavior adolescent"[ALL] OR "Adolescent Behaviors"[ALL] OR "behaviors adolescent"[ALL] OR "Child"[ALL] OR "Children"[ALL] OR "Minor"[ALL] OR "Minors"[ALL]) AND ("Emotional dysregulation"[ALL] OR "Emotion dysregulation"[ALL])))
