# Supplementary material for: Moderators and mediators of the relationship between parental depression and children’s emotion dysregulation: a systematic review
Source: Front Psychiatry. 2025 Jul 8;16:1605718. doi: 10.3389/fpsyt.2025.1605718 (PMC12279834; doi:10.3389/fpsyt.2025.1605718)
Supplement: Supplementary file 2 [file Supplementaryfile2.docx]

| **Study Author** | **Number of Parents &and Youth Included**  **Timing of Depression Assessment** | **Study Characteristics**  **& Length of follow-up** | **Inclusion & Exclusion Criteria** | **Measures & Outcomes** | **Moderators & Mediators** | **Mediation/Moderation Analysis** | **Results** | **Notes** | **Funding Source**  **Risk of Bias (ROB)** |
| --- | --- | --- | --- | --- | --- | --- | --- | --- | --- |
| **(Felton et al. 2021)** | 206 adolescent - **mother** dyads  **Timing of depression Assessment**:  At every assessment.  T15 through T18 | Sample drawn from final four waves of a longitudinal study  Wave 5 is the baseline for this study, at which time average mother age was 46 years old (range:30-59) and average adolescent age was around 15 years old (range:13-17)  Follow up over 4 years (T16,17,18,19) | **Inclusion Criteria**  (1)Youth 9–13 years old  (2) proficient in English  (3) willing to participate in annual assessment.  **Exclusion Criteria:**  Not specified | **Measures**  **- Demographics:** Mothers reported their own and their adolescent’s age, grade, sex, and  race at each time point.  **- Maternal Depression 🡪** Center for Epidemiological Studies- Depression (CES-D)  **-Adolescent Depression 🡪** Center for Epidemiological Studies- Depression Child: CES-D  **-** **Maternal Emotion Regulation:** 36-item Difficulties in Emotion Regulation Scale  **- Adolescent Emotion Regulation (Youth Distress)** 🡪 The Behavioral Indicator of Resiliency to Distress (BIRD) | **Moderators**  Severity of baseline maternal depression | Structural Equation Model | Among the girls’ model, baseline levels of depressive symptoms, maternal Emotion regulation, and maternal depressive symptoms predicted changes in Distress Tolerance at T16 | Adolescent-mother sample drawn from longitudinal study  investigating the development of risky behaviors.  Study used Distress Tolerance as a proxy measure of Emotion Regulation in adolescents, as measured by BIRD (Youth Distress) | National Institute on Drug Abuse  ROB: Good |
| **(Marino et al. 2019)** | 104 infants (53 males, 51 females) & **Mothers**  **Timing of Depression Assessment:**  Postnatally & two weeks prior to the 6 months of age of their child | Child assessments at 6 &24 months of age | **Inclusion Criteria**  (1) both parents native-Italian speakers  (2) birthweight ≥ 2500 grams;  (3) APGAR scores at both 1 and 5 min ≥ 8  (4) a Bayley Cognitive Score ≥ 7;  (5) Absence of certified diagnosis of intellectual deficiency or autism spectrum disorder in first-degree relatives.  **Exclusion criteria**  Not specified | **Measures:**  **-Maternal Postnatal Depression ->** Achenbach System of Empirically Based Assessment  **Emotion Dysregulation ->** CBCL-DP  **-FAA & PAA were measured by** EEG  **- Potential Confounders:**  infant’s gestational weeks at birth, parental age, parental education and socioeconomic status, maternal and paternal education  **-Socioeconomic status:** Hollingshead 9-point scale | **Mediators**  Parietal alpha asymmetry (PAA): Mediation  Frontal alpha asymmetry (FAA): No mediation | Structural equation modeling | Greater levels of maternal depression symptoms predicted left parietal alpha asymmetry, which predicted higher levels of child emotion dysregulation. |  | Italian Ministry of Health and "5 per mille" funds for biomedical research.  ROB: Good |
| (Pina-**Camacho et al. 2015**) | 7814 **mother**–child pairs  **Timing of Depression Assessment:**  Prenatally (at 18 & 32 weeks of gestation), and four times after birth (at 8 weeks, 8 months, 2 years and 3 years).  For the current analysis, two latent depression scores were created: one for the prenatal period and one for the postnatal period. | Children followed until the age of 7 years old | **Inclusion Criteria**  Mothers who completed the SDQ  assessment at child age of 7 years  **Exclusion Criteria:**  Mother–offspring pairs who had no data  on the SDQ at child age of 7 years | **Measures:**  **-Demographics 🡪** pregnancy and birth information  **-Maternal depression ->** Edinburg Postnatal Depression Scale (EPDS) Twice prenatally (at 18 and 32 weeks of gestation), and four times after birth (at 8 weeks, 8 months, 2 years and 3  years).  **-Unhealthy Diet 🡪** Food Frequency Questionnaire (FFQ) prenatally (Mother’s diet)  Ages 3 & 4.5 years (Child Diet)  **-Child Emotional-Behavioral Dysregulation 🡪** Strengths and Difficulties Questionnaire (SDQ-DP) at Ages 2, 4, 7 years old.  **-Main outcome:** SDQ-DP at age 7 years | **Mediators:**  Unhealthy diet at age 3 years old |  | Unhealthy diet at the age of 3 years was a mediator of the association between maternal prenatal depression and emotion dysregulation at the age of 7 years. | Results drawn from the later Avon Longitudinal Study of Parents and Children.  Of the original 14 541 mother–child pairs, 7814 singleton mothers who completed the SDQ assessment at child age of 7 years were included. | UK Medical Research Council and the Wellcome Trust (grant reference 092731) and the University of Bristol provide core support for ALSPAC.  This research was specifically funded by the Eunice Kennedy Shriver National Institute of Child Health & Human Development of the National Institutes of Health (award number R01HD068437 to E.D.B  ROB: Good |
| **(Babineau et al. 2015)** | 213 **mother**-child pairs from MAVAN project.  **Timing of Depression Assessment**:  Prenatally at gestation age 24 & 36 weeks  Postnatally at 6, 12, 24 and 36 months  . | Women were recruited between 2003 and 2009 during routine ultrasound examinations in maternity hospitals.  Follow up at 6 months, at 18 months, and 36 months. | **Inclusion Criteria**  (1) Mothers age 18 years (2)Term pregnancy (≥ 37 weeks).  (2)Dyads with complete measures at 36 months  **Exclusion criteria**  (1)Presence of severe chronic maternal illness  (2) Past obstetrical complications  (3)major fetal/infant anomaly. | **Measures:**  **Postnatal Depression 🡪**  CES-D  **Prenatal Depression 🡪** CESD-D  **Emotion Dysregulation 🡪** IBQ-R complete by mothers when child is aged 3 and 6 months -  Early Childhood Behavioral Questionnaire (ECBQ) completed by mothers at ages 18 and 36 months  **Child and mother genotype for 5-HTTLPR 🡪** buccal swabs, using the standard TaqMan method on the ABI-7000 for Single Nucleotide Polymorphism markers and on the ABI-3100 for repeat polymorphisms.  **Covariates** 🡪 Health and Well Being of Mothers and their Newborns questionnaire administered prenatally and at 6, 12, 24 and 36 months postnatal, maternal education prenatally assessed | **Moderator**  Child’s 5-HTTLPR genotype | Mixed-model and confirmatory analyses | There was a significant interaction effect between prenatal depression and infant/child 5- HTTLPR on the outcome of dysregulation at 3, 6, 18 and 36 months (β = −.11, SE = .04, p < .01).  Carriers of the LALA genotype were insensitive to prenatal depression exposure, with stable scores of dysregulation throughout. Carriers of the S/LG genotypes, had higher levels of dysregulation as a function of exposure to greater levels of prenatal depression. With lower prenatal depression, S/LG carriers had lower levels of dysregulation than LA carriers. | Sample drawn from the longitudinal Maternal Adversity,  Vulnerability and Neurodevelopment (MAVAN) project.  Initial sample size 578 women. | Funded by Canadian Institutes of Health Research , Fonds de recherche du Québec  and the March of Dimes Foundation.  ROB: Good |
| **(Gonzalez 2009)** | 132 mother-toddler dyads  N= 66 mothers with a history of depression  N=66 mothers without depression or any psychiatric disorder  **Timing of Depression Assessment**:  At toddlers' twenty-  months of age | Study included a control group  Follow ups at children’s ages of 36 and 48 months  “Strange Situation” was performed on toddlers at the age of 20 months (Time 1) and age 36 months (Time 2). | **Inclusion criteria**  (1)parents with at least a high school education  (2) parents not reliant on public assistance.  (3)-mothers had been diagnosed with MDD and have had an episode of depression (but not necessarily their first episode of MDD) at some time after the birth of their child and had a child of approximately 20 months of age.  **Exclusion Criteria**::  -Mothers who met criteria for bipolar disorder *(N=* 8)  **Control group Inclusion criteria:**  Mothers who lived in same vicinity as the families of mothers with a history of depression.  **Control Group Exclusion Criteria:**  Participants who presented current or past symptomatology of any major psychiatric disorder | **Measures:**  **-Emotion Dysregulation 🡪** *Tool use and Problem-Solving Task* at age 48 months  **-Maternal affective discourse 🡪** *Infant Facial Expressions of Emotion from Looking at Pictures (IFEEL)*  **-Depression 🡪** History of depression. Diagnostic Interview Schedule m-R was done with mothers to assess present or past history of MDD and of any psychiatric disorder  **-Mother-Toddler Attachment Organization 🡪** Strange Situation paradigm at toddler’s age 20 and 36 months | **Mediators:**  Maternal affective discourse: (no mediation)  Toddler’s degree of attachment security: (no mediation ) | Structural Equation Modeling (SEM) framework.  Test of intervening variables | Maternal depression at Time 1 was significantly linked to attachment insecurity at Time 1, (r = . 15, p< .000) and significantly predicted toddlers' attachment insecurity at 36-months (P = .28, p<03)  Maternal depression at Time 1 was significantly linked to affective discourse at Time 1, (r = - .03, p = .05) and significantly predicted affective discourse at Time 2 (P = -.16, p = .03).  Maternal affective discourse was not significantly associated with toddlers' emotion dysregulation problems at 48-months  . | Depression was not measured by a scale.  Study employed a control group | Funding: None specified  ROB: Poor |
| **(Lunkenheimer et al. 2021)** | 100 **mothers, fathers**, and 3 1⁄2-year-old children  **Timing of Depression Assessment:**  T1, at child’s age 41 months. | Average child age was 41 months (SD = 3 months) at (T1) and 45 months at follow-up (T2). | **Inclusion Criteria:**  Not specified. Families were recruited through flyers and email LISTSERVs at daycares, preschools, and agencies for families with young children  **Exclusion Criteria:**  Developmental delay or cardiac condition that would prevent physiological data collection. | **Measures:**  **-Parental Depressive Symptoms 🡪** CES-D  **-Emotional negativity/lability (Dysregulation at T2) 🡪** 24-item Emotion Regulation Checklist  **-Child Externalizing problem**s 🡪 Behavioral dysregulation at T2 as measured by Child Behavior Checklist (CBCL)  **-Dyadic Behavioral variability and dyadic positive behavior** > Gridware 1.15 and state space grids (SSG)  **-Observational coding** > All parents and child behaviors were recorded | **Moderator**  Parent-Child Dyadic Behavioral Variability | PROCESS 3.4 macro in Statistical Package for the Social Sciences 25 | At higher levels of paternal depressive symptoms, higher mother-child DBV predicted greater emotional negativity/lability in children, b = 0.37, t(70) = 2.08, p < .05.  When Maternal depressive symptoms were higher (1 SD above the mean), higher levels of mother-child DBV predicted greater child negativity/lability, b = 0.37, t(77) = 2.27, p < .05 | Dyadic positive behavior was operationalized as the total duration of mutually  positive behavior. For example, when the mother  engaged the child in a positive activity and the child complied. Lower positive content reflected higher negative content.  Eleven mothers and 10 fathers met criteria for clinical depression | National Institute of Child Health and Human Development of the National Institutes of Health (NIH), K01HD068170  National Science Foundation (NSF)    ROB: Fair |
| **(Fredriksen et al. 2019)** | 1036 families  **Timing of Depression Assessment:**  Prenatally & postnatally (T1 through T7) | Data from eight collection waves  Postnatal data collection waves at child age 6 weeks postpartum  (T5, n = 930), 6 months postpartum (T6, n = 860), 12 months postpartum (T7, n = 762), and 18 months postpartum (T8, n = 777) | **Inclusion Criteria:**  Pregnant women receiving prenatal care at nine selected well-baby clinics..  **Exclusion criteria:**  There were no exclusion criteria | **Measures:**  -**Maternal depression** 🡪 Edinburgh Postnatal Depression Scale (EPDS)  -**Parenting Stress** 🡪 Parenting Stress Index (PSI), at child’s age 12 months old.  -**Cognitive and Language Development** 🡪 Bayley Scales of Infant and Toddler Development, Third Edition at children age 18 months  **-Emotion Dysregulation** 🡪 The Infant-Toddler Social and Emotional Assessment (ITSEA ) | **Mediation**  Maternal parenting stress  **Moderation**  No moderation by Parents symptoms load | Mplus version 7.3 | The relation between maternal post- natal depression and dysregulation problems was mediated by maternal parenting stress at 12 months (b = 0.02, 95% CI [0.01, 0.03]; β = 0.22, 95% CI [0.15, 0.30]) | Families drawn from a larger prospective, multisite “Little in Norway study” | Research Council of Norway (Grant #196156 )  ROB: Good |
| **(Hoffman et al. 2006)** | Sample drawn from multisite longitudinal study  **Mothers & Fathers**  20% of families were single parent.  **Timing of Depression Assessment**  At time 0 | 3-year-old children ( n-208) followed to age 4 (*n* = 201) and their families  128 typically developing children  80 children with developmental delays  **Time 0**: Children aged 30-36 months  **Time 1**: Children aged 35-37 months old  **Time 2:** Children ages 47-49 months old | **Inclusion Criteria**  Not specified. Families were recruited from family resource centers and early intervention programs (e.g Infant Evaluation Program in Pennsylvania and the Head Start program in California); as well as from preschools and day care centers in both states.  **Exclusion criteria**  Neurological impairment (e.g., cerebral palsy), autism, and history of abuse. | **Measures:**  **-Demographic information 🡪** (family members ‘ethnicity, socioeconomic status, education level, marital status, health history  **-Maternal Depression 🡪** CES-D at time 0  **-Child Assessment**  **🡪** CBCL at time 0; Bayley Infant Scale of Development at time 0.  **-Emotion Dysregulation -🡪 T**he dysregulation coding system at time 2; children ages 47-49 months; through three problem solving tasks, clean-up task, wait task, creating a composite index.  **-Maternal Scaffolding 🡪** Measured at Time 1. Structured Laboratory Visit to measure scaffolding effectiveness through three problem solving tasks and a clean-up task, creating a composite index | **Mediation**  Maternal Scaffolding (No mediation) | Hierarchical multiple regression | Mothers with high depressive symptomology are more likely to be ineffective at providing scaffolding for their children, and this lack of effective scaffolding increases the likelihood of child dysregulation. | Effective scaffolding involved a  mother providing the optimal level of support and assistance necessary to  allow her child to succeed beyond what she or he would have been capable  of achieving alone  To create an index of child dysregulation, children’s scores were averaged across five tasks, yielding one summary dysregulation score, which ranged from 0.00 to 3.80  Sample drawn from an ongoing multisite longitudinal study; 57 families were located in central Pennsylvania, and the other 151 families from southern California | National Institute of Child Health and Human Development  ROB: Fair |

**Appendix 2.** Detailed Data Extraction Sheet
